# Managing neurogenic orthostatic hypotension with droxidopa in a patient with Parkinson disease, atrial fibrillation, and hypertension

**DOI:** 10.1007/s10286-017-0433-7

**Published:** 2017-06-20

**Authors:** Ali Mehdirad, Beverly Karabin, Fiona Gupta

**Affiliations:** 1 Cardiac Electrophysiology Section, Division of Cardiology, Department of Internal Medicine, Saint Louis University, St. Louis, MO USA; 2 Syncope and Autonomic Disorders Center, University of Toledo, Toledo, OH USA; 3 Movement Disorders Center, Hackensack University Medical Center, Hackensack, NJ USA

**Keywords:** Atrial fibrillation, Hypertension, Neurogenic orthostatic hypotension, Parkinson’s disease, Droxidopa

## Challenge questions

How does supine hypertension influence treatment with droxidopa in patients with neurogenic orthostatic hypotension? What antihypertensive agents, if any, are appropriate for supine hypertension?

## Case presentation

Mr. Y is a 70-year-old man with Parkinson disease (PD) currently taking carbidopa/levodopa 25 mg/100 mg orally three times daily (TID). He has been living at home with his wife of 50 years, can ambulate independently, but has experienced three falls during the last 3 months.

He was referred to cardiology for evaluation of syncope. Reportedly, the patient had been in good health. After finishing a long dinner in a restaurant, he stood up to leave. His wife reported that he appeared somewhat dazed, and subsequently “crumbled to the floor”. He briefly lost consciousness (<30 s), came to without any neurological deficit, and he sustained no injury. He reported no prodromal symptoms of dizziness, lightheaded, or nausea, and did not appear pale.

An echocardiogram 1-year prior demonstrated mild left ventricular hypertrophy, but no systolic or diastolic dysfunction and no evidence of valvular disease. Carotid ultrasound 2 years prior revealed minimal carotid disease and no significant stenosis. His past medical history is significant for atrial fibrillation and a transient ischemic attack (TIA). He is currently taking metoprolol XL 25 mg/day and aspirin 325 mg/day.

The patient was determined to have a CHA_2_DS_2_-VASc [4] score of 5 (age, hypertension, history of TIA, and carotid disease all contributing to this score), a high-risk status for stroke and systemic embolism for which he was initially prescribed apixaban 5 mg twice daily. He was also taking aspirin 81 mg daily due to the presence of carotid artery disease. However, unfortunately, as a result of frequent falls and concerns for development of injuries (e.g., subdural hematoma), after a thorough discussion with the patient and his family about the benefits and risks of anticoagulation in the patient, apixaban was discontinued and instead aspirin dosage was increased to 325 mg daily.

## Expert commentary (Dr. Mehdirad)

Though several studies have demonstrated that aspirin therapy alone may be helpful in this setting, it is definitely not a replacement for anticoagulation for stroke prevention in patients with CHA_2_DS_2_-VASc score of 2 or higher. However, if this patient’s neurogenic orthostatic hypotension (nOH) symptoms, including his falls, can be successfully managed with droxidopa, he will again become an excellent candidate for resumption of anticoagulation (apixaban) along with aspirin 81 mg daily.

## Case continuation

The office evaluation demonstrated a seated blood pressure (BP) of 148/85 mmHg with a heart rate (HR) of 76 beats per minute (bpm). His BP after 3 min of standing was 102/68 mmHg with an HR of 82 bpm. He was symptomatic upon standing and reported feeling “a little dizzy”. On the basis of his medical history and his office BP and HR, the diagnosis of symptomatic nOH was made.

## Expert commentary (Dr. Gupta)

Working across specialties is imperative for optimal outcomes of nOH. This patient has nOH with a significant impact on quality of life. As a neurologist, I conduct a full history and examination to rule out associated comorbidities in patients with nOH, such as PD or other movement disorder as it was the case in this patient. However, given this patient’s history of underlying cardiac issues and current medication regimen, I would also consult with his cardiologist to convey my thoughts and concerns, and ascertain whether further cardiac workup is warranted. Again, a team approach is imperative and necessary for a successful treatment of nOH in patients with cardiac comorbidities, like this one.

## Case continuation

The patient was initiated on droxidopa at a dose of 100 mg on a modified TID schedule (taken on awakening/before rising from bed, at midday, and at least 3–4 h prior to bedtime) [1, 3]. During titration of droxidopa by 100 mg every 24–48 h, the patient and his wife were instructed to monitor his BP at home for at least 2 weeks during titration. They were instructed to measure BP/HR first thing in the morning in the supine position before morning medications and then standing for 3 min after arising from bed. In addition, they were asked to monitor BPs/HRs when the patient felt symptomatic, and at bedtime (approximately at least 15 min after lying down at bedtime). After titrating droxidopa by 100 mg every 24–48 h, the patient reached a droxidopa dose of 600 mg on the modified TID schedule. At that time, his wife, who had been keeping the BP log, reported that his BP readings during the day had been within the normal range; however, at night, they were noticeably high. The patient’s BP log revealed that the average BP at night was 165/92 mmHg. This was noted to be supine hypertension (sHTN) likely related to the patient’s nOH and, perhaps, aggravated by the administration of droxidopa.

At follow-up, the patient was instructed to sleep with the head and torso elevated at least 30–45°. It was explained to the patient and his wife that using an electric bed or mattress was the most effective way of accomplishing this, although other approaches (placing a wedge under the mattress or blocks under the legs of the bed’s headboard) could be used. In addition, the patient was instructed to avoid a supine posture during the day and preferably use a head-up recliner when resting or napping. The patient was advised to incorporate conservative non-pharmacologic measures, such as use of compression garments and abdominal binders during the day and removing them at bedtime, keep adequately hydrated, and have a snack at bedtime, which can also help to reduce sHTN. To further manage his sHTN at night, the patient was prescribed a short-acting antihypertensive agent, captopril 2.5 mg, at bedtime.

## Expert commentary (Dr. Mehdirad)

In this case, it is important to educate both the patient and his wife that nOH on its own or in conjunction with droxidopa therapy is the probable cause for sHTN [2, 5]. However, a mild supine BP elevation (such as this patient’s BP readings) is not uncommon and is, in fact, expected; hence, frequent and meticulous BP measurement is not usually needed. Furthermore, the evening dose of droxidopa should be taken at least 3–4 h prior to the patient’s bedtime. Volume-depleting agents, such as diuretics, and long-acting antihypertensive agents are not warranted in this patient, as he requires a reduction in BP only at nighttime. If elevating the head of the bed 30–45° is not enough to reduce supine hypertension, short-acting agents such as captopril 2.5 mg at bedtime are a good option. It should be stressed that too aggressive treatment of sHTN may lead to an increased frequency of syncope and falls as a result of more severe OH upon standing in the middle of the night to go to the bathroom, or upon getting up the next morning. In this particular case, metoprolol should be continued for ventricular rate control in the setting of atrial fibrillation.

## Case conclusion

After being on a steady droxidopa dosage of 600 mg TID, incorporating non-pharmacological measures, and adding a short-acting antihypertensive agent at bedtime, the patient and his wife now report that his daytime and evening BP readings have been within the normal range. Also, he reports not having had any falls or other symptoms of nOH over the past 2 months. As a result of his lack of falls, anticoagulation therapy can now be re-initiated.

## Expert commentary (Dr. Karabin)

For a patient such as Mr. Y who is experiencing sHTN, another option to consider is adjusting the dosage of droxidopa by administering a lower dose of droxidopa in the evening (the final dose of the day). If elevating the head of the bed 30–45° does not ameliorate supine hypertension, I would also consider comorbid conditions in determining the best-choice antihypertensive agent to administer at bedtime to treat sHTN associated with nOH in this patient as follows:

- Diabetes mellitus: consider a short-acting angiotensin-converting enzyme inhibitor at bedtime.
- Heart disease: continue the daytime beta-blocker also at bedtime.
- Insomnia: consider clonidine 0.1 mg at bedtime. This should be used with caution, as clonidine half-life is approximately 12 h, and there might be a hypertensive rebound after the effect weans off.

Again, providers must take into consideration that pharmacological treatment of sHTN can increase the frequency and severity of syncope and falls as a result of more severe nOH when the patient stands up in the middle of the night to go to the bathroom, or upon getting up the next morning.
